# Hydrocephalus and Diffuse Alveolar Hemorrhage as the Initial Manifestation of Systemic Lupus Erythematosus and Antiphospholipid Syndrome: A Case Report

**DOI:** 10.1002/ccr3.73543

**Published:** 2026-09-15

**Authors:** Ehsan Adib, Narges Gorbanmaleki, Amirreza Eterafi, Sara Jalali Jivan

**Affiliations:** ^1^ Cancer Immunology and Immunotherapy Research Center (CIIRC), Ardabil University of Medical Sciences Ardabil Iran; ^2^ Students Research Committee, School of Medicine Ardabil University of Medical Sciences Ardabil Iran; ^3^ Department of Internal Medicine, School of Medicine Ardabil University of Medical Sciences Ardabil Iran

**Keywords:** antiphospholipid syndrome, case report, diffuse alveolar hemorrhage, hydrocephalus, systemic lupus erythematosus

## Abstract

Systemic lupus erythematosus (SLE) is an autoimmune disease that can affect many systems, such as the central nervous system (CNS), cardiovascular, musculocutaneous, renal, and respiratory systems, etc. SLE can occur alone or in association with other autoimmune diseases, especially antiphospholipid syndrome (APS). Hydrocephalus and diffuse alveolar hemorrhage (DAH) are infrequent manifestations of SLE and APS and increase the risk of mortality and morbidity in these patients. A 48‐year‐old man was hospitalized due to fever, hemoptysis, and headache, which had not been examined for diagnosis and treatment for a long period of time because of his negligence. After imaging and autoimmune tests were performed, hydrocephalus and DAH were diagnosed as initial manifestations of SLE in association with APS according to the SLE/APS criteria. Additionally, lupus nephritis was confirmed via renal biopsy. The hydrocephalus was due to sigmoid sinus thrombosis in the context of a hypercoagulable state of SLE and APS. The initiation of steroid and immunosuppressive treatments has yielded positive results, negating the need for any invasive procedures. The coexistence of SLE and hydrocephalus, although uncommon, has been documented in the literature. Hydrocephalus, a rare complication of SLE, necessitates timely decision‐making regarding treatment based on the patient's clinical symptoms and the degree of ventricular dilation, which may involve either conservative medical management or surgical procedures such as a VP shunt. In patients with multiorgan involvement, rheumatological and autoimmune diseases are among our most important differential diagnoses. Importantly, neurological symptoms, particularly headaches, should not be underestimated, even though they are commonly observed in patients with SLE or APS. A detailed medical history, a comprehensive examination, and strong clinical suspicion are essential when patients present with neurological symptoms, and physicians must consider autoimmune diseases in their differential diagnoses.

## Key Clinical Message

This case highlights the importance of timely diagnosis and treatment of hydrocephalus and DAH as two rare manifestations of SLE and suggests that the initial presentation of SLE, or any other disease, may be a rare manifestation rather than a common manifestation.

**Abbreviations**
APA: Antiphospholipid antibody
APS: Anti‐Phospholipid Syndrome
BAL: bronchoalveolar lavage
BBB: blood–brain barrier
CKD: Chronic Kidney Disease
CNS: Central Nervus System
CRP: C Reactive protein
CSF: Cerebrospinal Fluid
CVST: Cerebral venous sinus thrombosis
DAH: Diffuse Alveolar Hemorrhage
GFR: Glomerular Filtration Rate
GPA: granulomatosis with polyangiitis
HBS Ag: Hepatitis B surface antigen
HCV Ab: Hepatitis C virus antibody
HIV: Human immunodeficiency virus
ISN/RPS: International society of nephrology and renal pathology society
LP: Lumbar Puncture
MRI: Magnetic Resonance Imaging
MRV: Magnetic Resonance Venography
NPSLE: Neuropsychiatric Systemic lupus erythematosus
PCR: Polymerase Chain Reaction
SCLE: Subacute cutaneous lupus erythematosus
SLE: Systemic lupus erythematosus
VP: Ventriculoperitoneal
VTE: Venous Thromboembolism

## 1 Introduction

Systemic lupus erythematosus (SLE) is a multisystem chronic autoimmune disease characterized by relapsing and remitting symptoms. The underlying causes of SLE are still not completely understood. Studies have demonstrated that the interplay between environmental and genetic factors can trigger immune responses that lead to excessive production of pathogenic autoantibodies by B cells, as well as cytokine dysregulation, contributing to tissue and organ damage. The disease affects an average of 50 individuals per 100,000 people, with a female predominance of 9:1, particularly in young women. SLE is characterized by inflammation and immune‐mediated damage across various organ systems, such as the mucocutaneous, musculoskeletal, hematologic, and renal systems. It presents with a diverse array of symptoms, including skin rashes, persistent fatigue, arthritis, glomerulonephritis, cardiovascular complications, strokes, and effects on the respiratory system. Many of these manifestations can contribute to early mortality [1, 2, 3].

Neuropsychiatric SLE (NPSLE) is a term that refers to neurological or psychiatric symptoms that occur specifically after other possible causes are excluded. The prevalence of manifestations of NPSLE is approximately 50%, which includes stroke, meningitis, movement disorders, myelopathy, psychiatric features, etc., and is associated with a poor prognosis since involvement of the central nervous system (CNS) is the second most frequent cause of mortality. Among its notably rare but dangerous neurological complications is hydrocephalus, whose underlying pathophysiology is not fully understood; thus far, only a few case series and reports exist [4, 5, 6, 7].

Herein, we present a case report of a 48‐year‐old male patient who, following fever, hemoptysis, and headache, was finally diagnosed with hydrocephalus, diffuse alveolar hemorrhage (DAH), and lupus nephritis as initial manifestations of SLE in association with APS.

## 2 Case Presentation

### 2.1 Presenting Complaints, Case History, and Clinical Examination

A 48‐year‐old male patient presented to the emergency department of our hospital with flu‐like symptoms, including fever, headache, and chills, along with intermittent cough, phlegm, and hemoptysis of spoon‐sized volume over the previous 3 days. He had been experiencing similar symptoms periodically for 2 years, but had not been investigated and had neglected them. In the patient's previous medical history, he had been suffering from thalassemia minor, with an average hemoglobin level of 11–12 for many years, and received packed blood cells on an outpatient basis. He had hypertension for 10 years, diabetes mellitus for 2 years, and hypothyroidism for approximately 10 years, all of which were under appropriate medical control. The patient also had unspecified mucocutaneous and psoriatic lesions on parts of his body for 10 years, which he had neglected to evaluate. No significant family history of disease was reported. His consciousness was clear, and he was fully alert and oriented to time, place, and person. The vital signs recorded included a blood pressure of 125/85 mmHg, a heart rate of 114 beats per minute, a respiratory rate of 22 breaths per minute, a body temperature of 38.9 degrees Celsius, and an arterial oxygen saturation of 94%. The conjunctiva appeared pale, and both pupils responded to light. The oral mucosa was well‐hydrated and showed no signs of ulcers. Upon skin examination, there were no signs of petechiae, purpura, or ecchymoses. On cardiac auscultation, no pathological sounds were heard. Additionally, during the lung examination, crackles and decreased breath sounds were heard at the bases of both lungs. No deformity or specific problems were observed in the joints or limbs; cranial nerve examination was normal, and there was no sensory deficit.

### 2.2 Investigations

Initial hematology, serology, and biochemistry tests were performed on the patient, and the results are presented in Tables 1 and 2. Tests for *Mycobacterium tuberculosis* , influenza, COVID‐19, HBV, HCV, and HIV were also performed; all of the tests were negative. Blood and urine cultures were also performed, and no microbial growth occurred. A CT scan of the head, neck, and chest was also performed on the patient. The chest X‐ray and electrocardiogram results were normal.

**TABLE 1:** Hematology results.

Hematology parameter's	Result	Normal range
WBC (10*3/μL)	7.0	4–10
RBC (10*6/μL)	4.5	4.2–5.4
HGB (g/dl)	8	12–16
HCT (%)	28	36–46
MCV (FL)	62	81–96
MCH (pg)	18	26–32
MCHC	28	26–36
Platelets (10*3/μL)	169	150–450
ESR 1st h (mm/h)	90	0–15
P.T (Sec)	12	—
INR	1.07	—
P.T.T (Sec)	30	30–45

Abbreviations: ESR, erythrocyte sedimentation rate; HCT, hematocrit; HGB, hemoglobin; INR, international normalized ratio; MCH, mean corpuscular hemoglobin; MCHC, mean corpuscular hemoglobin concentration; MCV, mean corpuscular volume; PT, prothrombin time; PTT, partial thromboplastin time; RBC, red blood cell, RDW‐CV, red cell distribution width; WBC, white blood cell.

**TABLE 2:** Blood biochemistry results.

Biochemical parameters	Result	Normal range
Blood glucose (mg/dl)	90	70–100
Urea (mg/dl)	61	10–50
Creatinine (mg/dl)	2.0	Male (0.9–1.3)
K (Potassium) (mEq/L)	3.5	3.5–5.5
Na (Sodium) (mEq/L)	146	132–145
Mg (Magnesium) (mg/dl)	1.9	1.8–2.6
Ca (Calcium) (mg/dl)	10	8.2–10.7
AST (IU/L)	13	5–40
ALT (IU/L)	16	5–40
Alk.P	155	64–306
Total Bili	0.6	0.2–1.2
Direct Bili	0.17	0–0.4
Albumin	4.3	3.5–5.5
LDH	623	0–500
Iron	73	70–175
TIBC	175	230–440

Abbreviations: Alk.P, alkaline phosphatase; ALT, alanine transaminase; AST, aspartate transaminase; Bili, Bilirubin; CPK, Creatine phosphokinase; LDH, lactate dehydrogenase; TIBC, total iron‐binding capacity.

**FIGURE 1:**
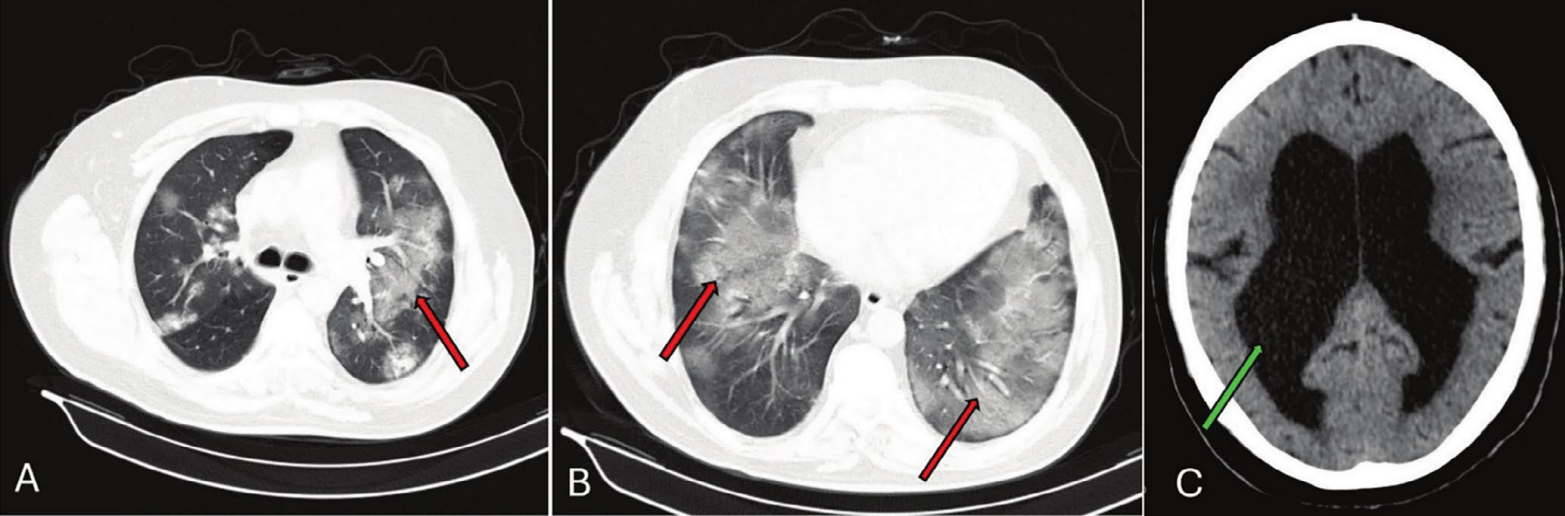
Ground‐glass opacities and patchy consolidation throughout the parenchyma of both lungs, which indicate DAH in the lung CT scan (A&B, red arrows). Ventriculomegaly and hydrocephalus in the brain CT scan (C, green arrow).

**FIGURE 2:**
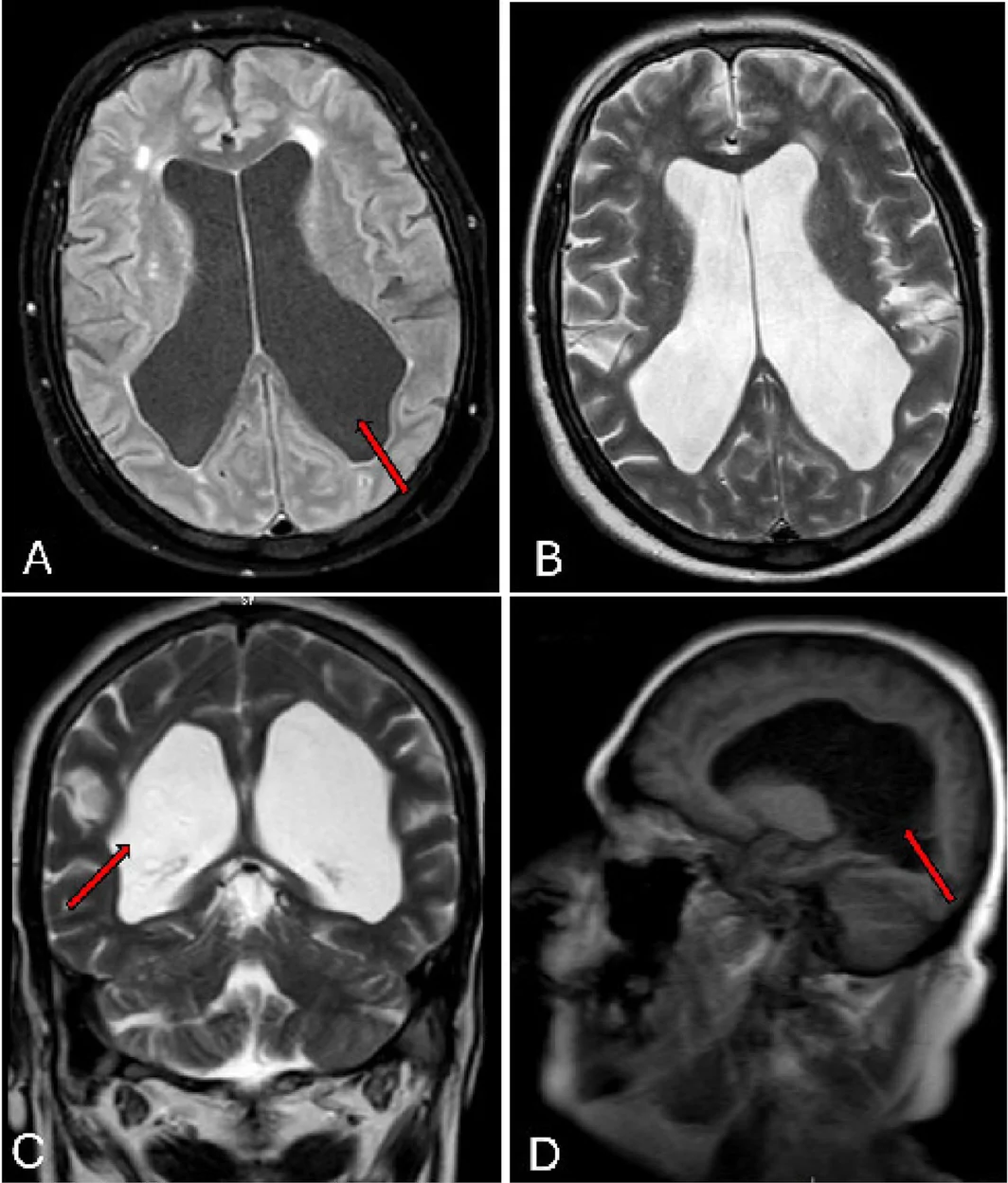
T1(A) and T2(B)‐ weighted brain MRI show moderate to severe two‐ventricle hydrocephalus (both lateral ventricles) in transverse (A and B), coronal (C), and horizontal (D) views (red arrows).

According to the patient's initial test results, the patient's creatinine increased (Cr = 2, GFR: 52), and he also had proteinuria of 370 mg in 24 h, indicating nephropathy. The Hb level was low (= 8), and the ESR was also elevated (= 90). Two units of packed blood cells were also transfused into the patient, which increased his hemoglobin to 9.5. A CT scan of the chest revealed ground‐glass opacities and patchy consolidation throughout the parenchyma of both lungs. Given the patient's decreased hemoglobin, hemoptysis, and CT findings, the evidence was primarily in favor of DAH. Therefore, bronchoalveolar lavage (BAL) was performed, which revealed progressively hemorrhagic aliquots with bronchial epithelial cells, alveolar macrophages, and inflammatory cells. It was also negative for malignancy, bacterial and fungal cultures. Additionally, a CT scan of the head and neck revealed ventriculomegaly and hydrocephalus (Figure 1). To further evaluate the hydrocephalus, a brain MRI was performed. MRI images revealed the same findings as the CT scan and revealed moderate to severe two‐ventricle hydrocephalus (both lateral ventricles), and multiple high signal intensity small foci on long TR images in the periventricular white matter (microvascular ischemic changes) were observed (Figure 2). Lumbar puncture revealed normal pressure, cell counts, protein levels, and glucose levels.

On the basis of the results of the tests and imaging, the presence of fever and an increased ESR (= 90), lung involvement in the form of DAH, renal involvement in the form of increased creatinine, and suspicion of systemic autoimmune and rheumatological diseases increased. Therefore, an autoimmune workup was performed, and the results are shown in Table 3.

**TABLE 3:** Rheumatological and autoimmune test results.

	Result	Normal range
C3	1.14	0.9–1.8
C4	0.2	0.1–0.4
ANA	2.2	Negative < 1 Positive > 1
FANA	1/50	Negative < 1/80 Positive > 1/80
Anti beta2 glycoprotein (IgM)	6	Negative < 10 Positive > 10
Anti beta2 glycoprotein (IgG)	83.5	Negative < 10 Positive > 20
Lupus anti‐coagulant (LAC)	60	31–44
P‐ANCA	Negative	Negative < 1/10
C‐ANCA	Negative	Positive > 1/10
Anti‐ds‐DNA	105.6	Negative < 30 Positive > 50
Anti‐cardiolipin Ab (acL) (IgG)	99.1	Normal < 10 Elevated > 10
Anti‐cardiolipin Ab (IgM)	5.8	Normal < 7 Elevated > 7
Anti La	6.5	Negative < 10 Positive > 10
Anti G.B.M	3.2	Negative < 40 Positive ≥ 40

Abbreviations: ANA, antinuclear antibodies; C, complement; C‐ANCA, Cytoplasmic Antineutrophil cytoplasmic autoantibody; ds‐DNA, double‐stranded deoxyribonucleic acid; FANA, fluorescent antinuclear antibody; G.B.M; glomerular basement membrane; P‐ANCA, perinuclear anti‐neutrophil cytoplasmic antibodies.

### 2.3 Final Diagnosis and Treatment

Given the positive anti‐beta2 glycoprotein (= 83.5), ANA (= 2.2), anti‐ds‐DNA (= 105.6), and anti‐cardiolipin IgG (= 99.1) tests, a diagnosis of systemic lupus erythematosus (SLE) was initially considered for the patient. To be completely certain and confirm this diagnosis, the EULAR/ACR 2019 criteria were used [8]. According to these criteria, a score above 10 is considered positive, and since the score obtained for this patient was 14 (fever: 2, subacute cutaneous or discoid lupus: 4, positive anti‐cardiolipin Ab or anti‐beta2 glycoprotein Ab: 2, anti‐ds‐DNA: 6), the diagnosis of SLE was confirmed. With the diagnosis of SLE, the psoriatic‐like skin lesions in this patient can be considered lesions of subacute cutaneous lupus erythematosus (SCLE). Suspecting lupus nephritis due to elevated creatinine and proteinuria, the patient underwent a renal biopsy, which confirmed Class II (mesangial proliferative) lupus nephritis on the basis of the ISN/RPS classification, with mesangial hypercellularity without crescentic or segmental lesions or mesangial immune deposits.

Immediately after the diagnosis, the patient was transferred to the rheumatology service, and intravenous corticosteroid pulse therapy with methylprednisolone (1 g/day for 3 days) was administered. Maintenance methylprednisolone at 80 mg/d was then initiated. After consulting with the hospital neurologist, intravenous mannitol was given, followed by oral acetazolamide 250 mg twice a day. His condition began to stabilize during hospitalization.

**FIGURE 3:**
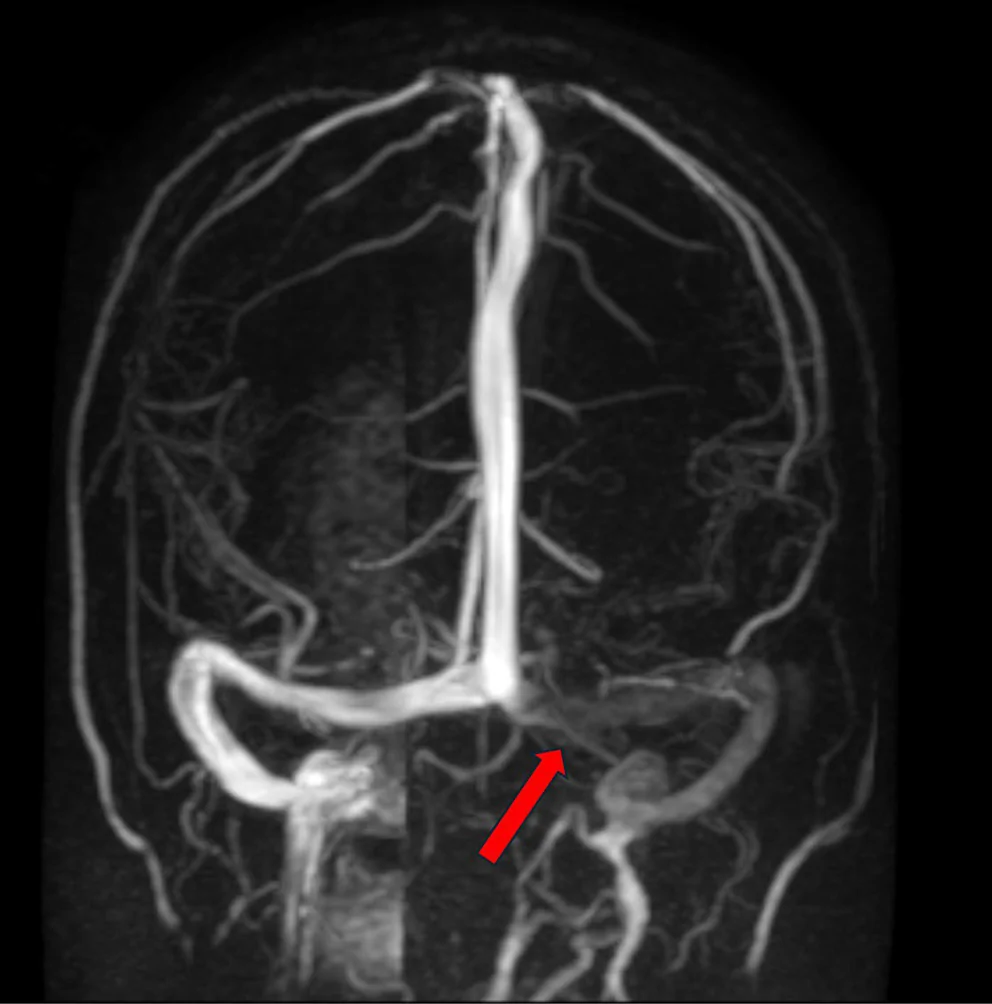
MRI venography image showing right transverse and sigmoid sinus thrombosis (arrow) with no flow in this vein.

Given the positive lupus anticoagulant antibody (LAC), anticardiolipin antibody (acL), and anti‐beta2 glycoprotein antibody results, the patient was suspected of having antiphospholipid syndrome (APS) in addition to SLE. However, given that this patient had no history of deep vein thrombosis (DVT) in the lower or upper extremities or pulmonary embolism in the past, the cause of this patient's hydrocephalus was likely chronic thrombosis in the cerebral vessels. Therefore, MR venography (MRV) was performed on the patient, which revealed cerebral venous sinus thrombosis (CVST) and absent flow in the veins of the right sigmoid sinus and right transverse sinus (Figure 3). Upon observing the presence of this thrombosis as well as the presence of the autoantibodies mentioned above, the possibility of APS in association with SLE was considered. To be completely certain and confirm this diagnosis, the EULAR/ACR APS 2023 criteria were used [9]. In these criteria, obtaining at least 3 points from the clinical domain and at least 3 points from the laboratory domain is classified as the APS. The score obtained for this patient in the clinical domain was 8 (VTE with a high‐risk VTE profile: 1, pulmonary hemorrhage (BAL or pathology): 5, pulmonary hemorrhage (symptoms and imaging): 2), and in the laboratory domain was 8 (positive LAC (single‐one time): 1, high positive (IgG) (acl and aβ2GPI): 7). Therefore, the diagnosis of APS was confirmed. Anticoagulant treatment with warfarin tablets was added to the patient's medications to prevent complications and possible thrombosis related to APS in the future.

### 2.4 Outcome and Follow‐Up

After a hospitalization period of 10 days, the patient was discharged from the hospital in good general condition with mycophenolate mofetil 500 mg twice a day as an immunosuppressive agent for maintenance treatment, hydroxychloroquine 200 mg daily, low‐dose (tapered to 15 mg/d) prednisolone, warfarin, supplemental drugs (folic acid, vitamin D, and calcium), and oral acetazolamide (for one month). He was advised to return to the rheumatology clinic within 4 weeks for reevaluation of treatment adequacy and disease course. After the patient returned, the autoantibody and ESR levels decreased, and his other laboratory parameters improved. The patient was completely satisfied with the improvement in his symptoms. The patient's headache had also decreased to a great extent, so with the advice of the neurologist, the patient's oral acetazolamide was discontinued. He did not need LP therapy or a ventriculoperitoneal (VP) shunt during regular follow‐up.

## 3 Discussion

In summary, we present a case of SLE and APS, which led to hydrocephalus caused by sigmoid sinus thrombosis in the context of a hypercoagulable condition associated with SLE and APS. The initiation of steroid and immunosuppressive treatment yielded positive results, eliminating the need for invasive procedures such as LP therapy or a VP shunt.

The interplay among blood–brain‐barrier (BBB) dysfunction, autoantibody‐mediated effects, direct neuronal and vascular damage, and other factors may contribute to the pathogenesis of diverse manifestations of NPSLE [10]. Hydrocephalus as a manifestation of NPSLE is uncommon, with only a few cases documented in the literature. In a review by Ahmed et al. analyzing 13 cases of hydrocephalus associated with SLE, headache was the most prevalent symptom reported before or during the development and diagnosis of hydrocephalus. Additional symptoms noted included focal neurological deficits, seizures, drowsiness, delirium, urinary incontinence, instability in gait, depression, cognitive impairments, and coma. Furthermore, CSF analysis is largely nonspecific and often fails to reveal inflammatory markers in most cases [11]. The specific mechanisms underlying hydrocephalus pathogenesis are often not well understood in many cases. Cerebral phlebitis, meningeal inflammation, leptomeningeal and cerebral vessel microthrombi, hypercoagulable states, microvascular thrombosis secondary to perivascular infiltration, the deposition of immunoglobulins and complements on the walls of dural vessels, deposition within the arachnoid villi, post‐inflammatory aqueduct stenosis, and SLE‐induced vasculitis with subarachnoid hemorrhage are among the pathological abnormalities found in some patients. These findings also highlight the role of inflammation in the pathogenesis of hydrocephalus [12, 13, 14, 15, 16, 17, 18]. Like our patient, a case of hydrocephalus involving a 24‐year‐old woman with SLE and APS, whose hydrocephalus was a consequence of her hypercoagulable state and cerebral venous thrombosis, was reported by Mortifee et al. [17]. Additionally, increased antiphospholipid antibody (APA) titers at medium or high levels, prior or recurrent NPSLE events, heightened SLE activity, or organ damage resulting from SLE activity are risk factors for the development of hydrocephalus in the context of SLE [18]. Another group of SLE patients who develop hydrocephalus as a result of CNS infections, which arise during treatment with corticosteroids and immunosuppressive therapies or from spontaneous infections, has been identified; these infections promote the production but hinder the reabsorption of CSF [7, 10]. In our patient, a normal CSF profile ruled out the possibility of infection‐induced hydrocephalus.

CVST occurs when a clot in the venous sinuses of the brain blocks blood flow, preventing the brain from draining and leading to reduced CSF absorption, followed by hydrocephalus and increased intracranial pressure. Risk factors that predispose patients to thrombosis include obesity, malignancy, pregnancy, the use of oral contraceptives, head trauma, infectious diseases, hematologic diseases (such as sickle cell anemia), inflammatory and autoimmune diseases, blood clotting disorders that cause thrombosis, etc. The symptoms of thrombosis can vary depending on its location. On the basis of imaging and clinical presentation, we propose that the hydrocephalus experienced by our patient was a consequence of hypercoagulability‐induced damage to the arachnoid villi and direct damage and thrombosis affecting small venous structures in the brain, which disrupted and obstructed the outflow of CSF. In our case, CVST was likely a contributing factor to the presentation of hydrocephalus. The etiology of CVST is unknown, but previous connections between hydrocephalus and CVST have been established [19].

Regarding the management of hydrocephalus induced by autoimmune disorders, several treatment modalities have been suggested. These include medical management, which may involve the use of medications or VP shunting. Steroids are considered the main medical management method for hydrocephalus resulting from an autoimmune disorder. Conversely, the results indicate that, compared with medical management, VP shunting has yielded a much more positive outcome [7, 20]. The procedure for shunt placement was not performed for our patient because of the absence of severe specific symptoms associated with hydrocephalus. Consequently, a regimen of medication and regular follow‐up appointments was deemed appropriate for this patient, which resulted in a suitable improvement.

## 4 Conclusion

The findings from this case suggest that SLE and hydrocephalus can coexist, although this is a rare occurrence, as noted in prior literature. Despite hydrocephalus being an unusual manifestation of SLE, timely treatment decisions must be made on the basis of the patient's clinical presentation and the degree of ventricular dilation, and medical management or surgical interventions, such as a VP shunt, should be chosen. In patients with multiorgan involvement, rheumatological and autoimmune diseases are among our most important differential diagnoses. Importantly, neurological symptoms, particularly headaches, should not be underestimated, even though they are commonly observed in patients with SLE or APS. A detailed medical history, a comprehensive examination, and strong clinical suspicion are essential when patients present with neurological symptoms, and physicians must consider autoimmune diseases in their differential diagnoses.

## Author Contributions

**Ehsan Adib:** conceptualization, methodology, writing – original draft, writing – review and editing, investigation. **Narges Gorbanmaleki:** writing – review and editing, writing – original draft. **Amirreza Eterafi:** writing – review and editing, writing – original draft. **Sara Jalali Jivan:** supervision, writing – review and editing, writing – original draft, funding acquisition.

## Funding

The authors have nothing to report.

## Ethics Statement

The work was carried out by the Ethics Committee of Biomedical Research, Ardabil University of Medical Sciences, which issued approval IR.ARUMS.REC.1404.033.

## Consent

Written informed consent was obtained from the patient for publication of this case report. A copy of the written consent is available for review by the Editor‐in‐Chief of this journal.

## Conflicts of Interest

The authors declare no conflicts of interest.

## Data Availability

The data that support the findings of this study are available from the corresponding author upon reasonable request.
